# The complete chloroplast genome sequence of *Populus koreana* (Salicaceae)

**DOI:** 10.1080/23802359.2020.1715291

**Published:** 2020-01-24

**Authors:** Li Ang, Hou Zhe

**Affiliations:** Key Laboratory of Southwest China Wildlife Resources Conservation (Ministry of Education), College of Life Science, China West Normal University, Nanchong, China

**Keywords:** *P. koreana*, chloroplast genome, phylogenetic analysis, genetic information

## Abstract

The complete chloroplast genome sequence of *Populus koreana* was characterized using Illumina pair-end sequencing. The chloroplast genome of *P. koreana* was 156,868 bp in length, containing a large single-copy region (LSC) of 84,976 bp, a small single-copy region (SSC) of 16,606 bp, and two inverted repeat (IR) regions of 27,643 bp. The overall GC content is 30.70%, whereas the corresponding values of the LSC, SSC, and IR regions are 64.6%, 69.2%, and 60.1%, respectively. The genome contains 131 complete genes, including 86 protein-coding genes (62 protein-coding gene species), 37 tRNA genes (29 tRNA species), and eight rRNA genes (four rRNA species). The neighbour-joining phylogenetic analysis showed that *P. koreana* and *Populus fremontii* clustered together as sisters to other *Populus* species.

## Introduction

*Populus koreana*, from the family Salicaceae and the section Tacamahaca in the genus *Populus*, plays an important role in beautifying the environment, ecological defense, and industrial application in the Northeast China. In Changchun city, it is mainly cultivated for urban afforestation. *Populus koreana* can adapt to different climates and environments owing to without anthropogenic influence and harbor a wealth of genetic variation. *Populus koreana* harbours high ecological and economic value and has high level of intraspecific genetic diversity (Callahan et al. 2013). Therefore, *P. koreana* is an excellent system for understanding genetic information and genome variation patterns (Neale and Antoine 2011). Moreover, we can develop conservation strategies easily when we understand the genetic information of *P. koreana*. In the present research, we constructed the whole chloroplast genome of *P. koreana* and understood many genome variation information about the species, which will provide beneficial help for population genetics studies of *P. koreana*.

The fresh leaves of *P. koreana* were collected from Changchun city (43°48′N, 125°19′E). Fresh leaves were silica-dried and taken to the laboratory until DNA extraction. The voucher specimen (XY001) was laid in the Herbarium of China West Normal University and the extracted DNA was stored in the −80 °C refrigerator of the Key Laboratory of Southwest China Wildlife Resources Conservation. We extracted total genomic DNA from 25 mg silica-gel-dried leaf using a modified CTAB method (Doyle 1987). The whole-genome sequencing was then conducted by Biodata Biotechnologies Inc. (Hefei, China) with Illumina Hiseq platform. The Illumina HiSeq 2000 platform (Illumina, San Diego, CA) was used to perform the genome sequence. We used the software MITObim 1.8 (Hahn et al. 2013) and metaSPAdes (Nurk et al. 2017) to assemble chloroplast genomes. We used *P. tremula* (GenBank: NC_027425) as a reference genome. We annotated the chloroplast genome with the software DOGMA (Wyman et al. 2004) and then corrected the results using Geneious 8.0.2 (Campos et al. 2016) and Sequin 15.50 (http://www.ncbi.nlm.nih.gov/Sequin/).

The complete chloroplast genome of *P. koreana* (GenBank accession number MN864049) was characterized using Illumina pair-end sequencing. The chloroplast genome of *P. koreana* was 156,868 bp in length, containing a large single-copy region (LSC) of 84,976 bp, a small single-copy region (SSC) of 16,606 bp, and two inverted repeat (IR) regions of 27,643 bp. The overall GC content is 30.70%, whereas the corresponding values of the LSC, SSC, and IR regions are 64.6%, 69.2%, and 60.1%, respectively. The genome contains 131 complete genes, including 86 protein-coding genes (62 protein-coding gene species), 37 tRNA genes (29 tRNA species), and eight rRNA genes (four rRNA species).

**Figure 1.**
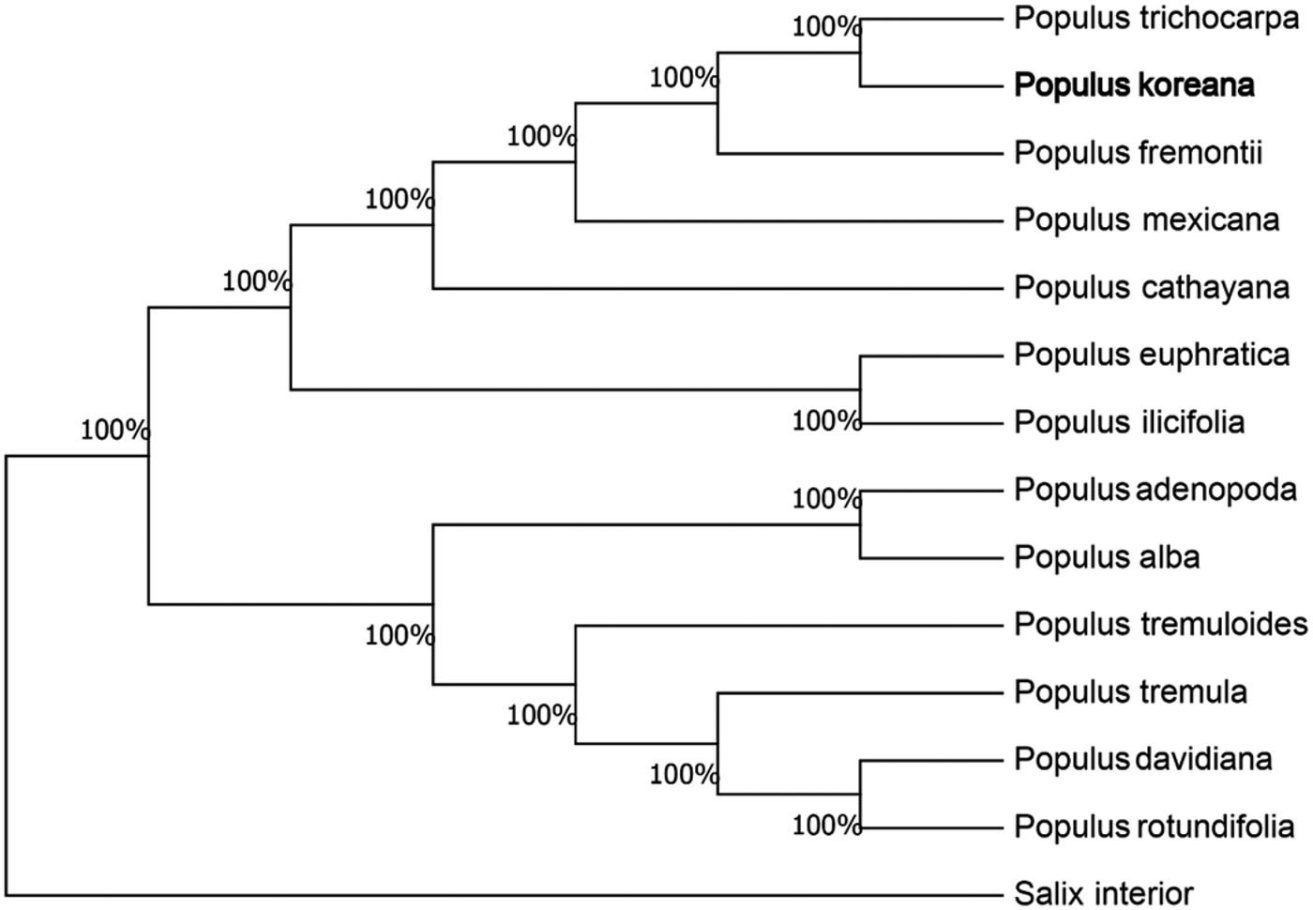
Neighbour-joining (NJ) analysis of *P. koreana* and other related species based on the complete chloroplast genome sequence. Genbank accession numbers: *P. tremula* (KP861984), *P. davidiana* (KX306825), *P. yunnanensis* (KP729176), *P. euphratica* (KJ624919), *P. adenopoda* (NC032368), *P. rotundifolia* (KX425853), *P. cathayana* (KP929175), *P. balsamifera* (KJ664927), *P. ilicifolia* (NC031371), *P. trichocarpa* (EF489041), *P. fremontii* (KJ664926), *P. tremuloides* (MN561844), and *Salix interior* (NC024681).

We used the complete chloroplast genomes sequence of *P. koreana* and 12 other related species of *Populus* and Salix interior as outgroup to construct phylogenetic tree. The 14 chloroplast genome sequences were aligned with MAFFT (Katoh and Standley 2013), and then the neighbour-joining tree was constructed using MEGA 7.0 (Kumar et al. 2016). The results confirmed that *P. koreana* was clustered with *P. trichocarpa* (Figure 1).
